# Decongested 2D IR Spectra Reveal N‑Terminal Helices in Amyloid Oligomers Associated with Type 2 Diabetes

**DOI:** 10.1021/jacs.6c16754

**Published:** 2026-09-21

**Authors:** Shivani T. Shivani, Josée Maurais, Brynn E. LeMasters, Harrison J. Esterly, Martin T. Zanni

**Affiliations:** Department of Chemistry, 5228 University of Wisconsin-Madison, Madison, Wisconsin 53706, United States

## Abstract

Oligomeric peptides are implicated in amyloidosis, yet only a few atomic structures of amyloid oligomers have been identified. Recently, we reported an atomic structural model of human islet amylin polypeptide (hIAPP) oligomer [ ShivaniS. T.,  Proc. Natl. Acad. Sci. U.S.A. 2026, 123, e2528103123 ]. The model was generated by trapping the oligomers with three alanine substitutions in a sequence termed 3A-hIAPP. These substitutions neither impact the physiologically critical β-sheet region of hIAPP nor the cytotoxicity and oligomer size measured through biochemical assays. However, their effect on the structure of the N-terminal helices could not be assessed. By modifying our 2D IR spectrometer, we interwove the acquisition of multiple pulse sequences, enabling waiting-time-dependent spectra of hIAPP oligomers to be measured during protein aggregation. Spectra acquired at a waiting time of 1.2 ps are decongested of disordered structures, revealing features originating from the oligomeric N-terminal α-helix and the adjacent kink and disulfide-loop regions. The spectra of hIAPP oligomers closely match those of 3A-hIAPP, strengthening the structural relevance of our atomic model for hIAPP oligomers. These findings also establish a workflow for testing and generating atomic structures of hIAPP oligomers associated with early-onset type 2 diabetes and for oligomers from other amyloid diseases.

## Graphical abstract

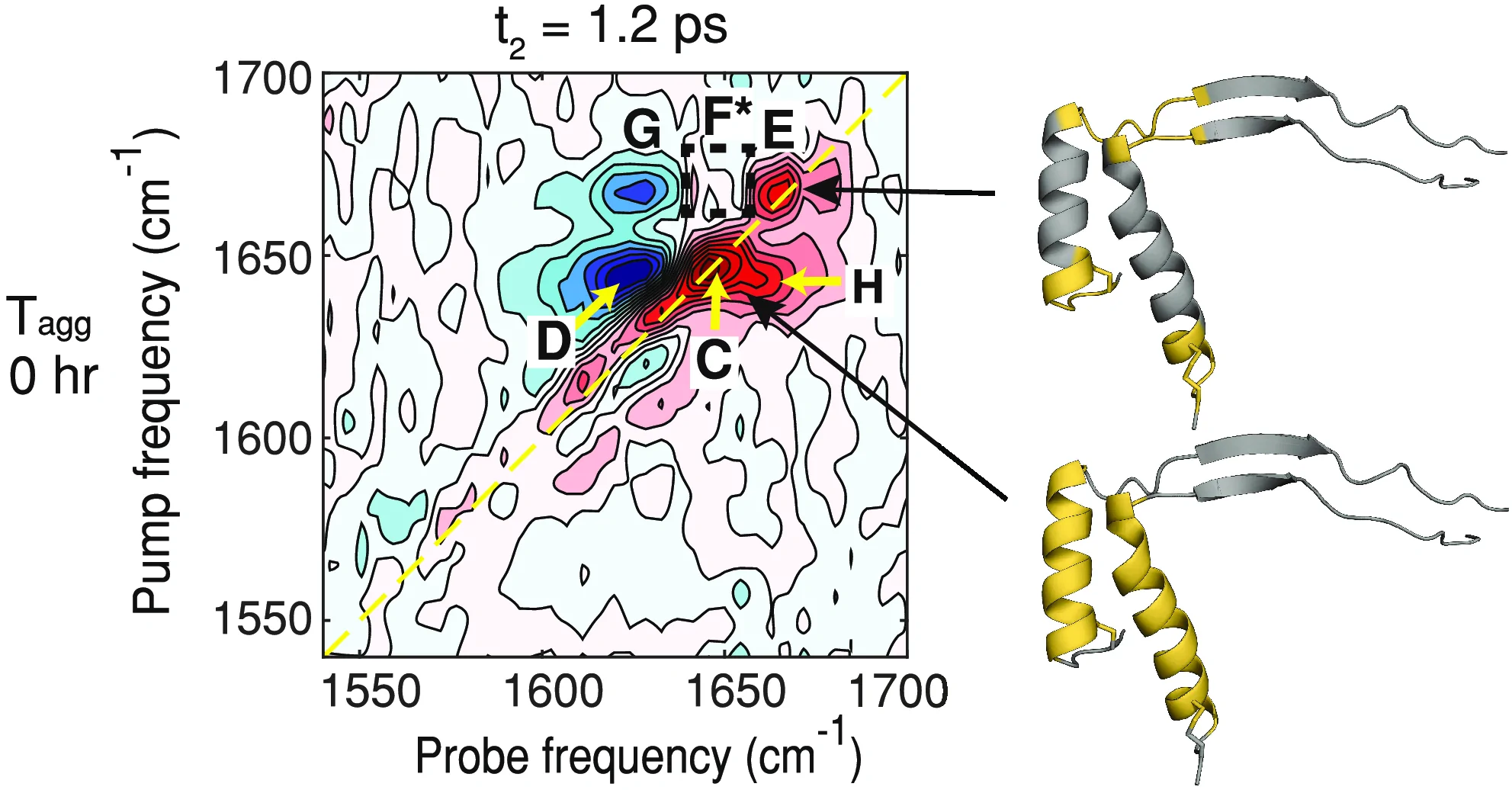

## Introduction

Intrinsically disordered or partially denatured proteins form transient oligomers prior to amyloid fibril formation, which constitute the disease-associated plaques (see Figure 1A for schematic). In many cases, the oligomers are thought to be a source of cytotoxicity, and they are notoriously difficult to structurally characterize due to their transient nature, low concentrations, small size, and heterogeneous size distributions.^1–5^ As a result, only a few atomic structures of amyloid oligomers have been reported.^6,7^ One approach to structural characterization has been to either stabilize the oligomers or destabilize the fibrils, thereby populating the intermediate oligomeric state by slowing the aggregation. This strategy has been implemented using antibodies, ligand binding, small-molecule inhibitors, cross-linking, and sequence truncations, followed by structural analysis using high-resolution techniques such as X-ray, cryoEM, and NMR spectroscopy.^6–14^ Although these methods have provided some of the earlier insights into the atomic structure of amyloid oligomers, it remains unclear whether the resulting oligomers represent native intermediate structures or are structurally altered.

**Figure 1:**
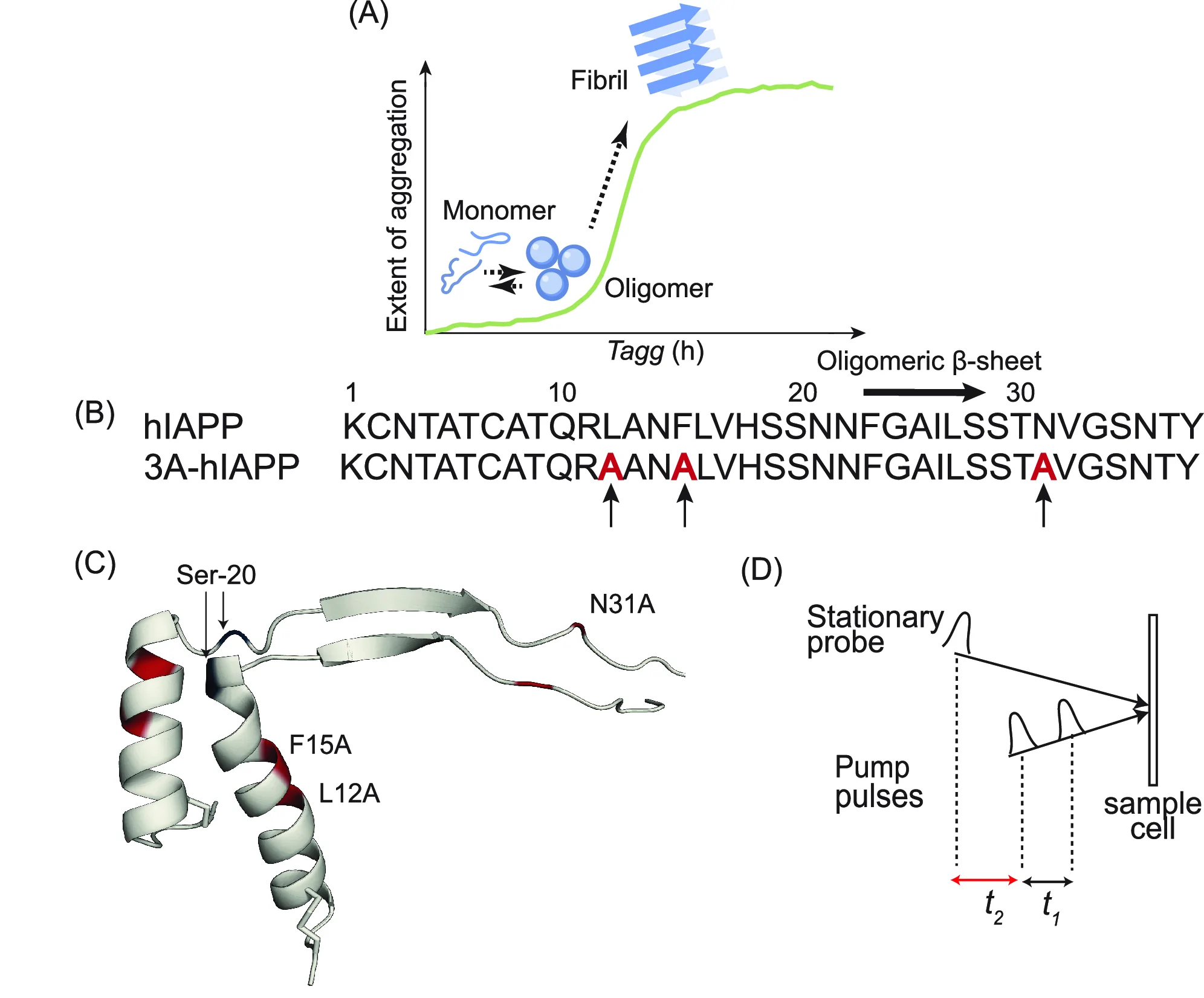
Schematic of aggregation mechanism, sequence of hIAPP and 3A-hIAPP, structure of model oligomer and pulse sequence showing coherence and waiting-time delays (denoted as *t*_1_ and *t*_2_). (A) Aggregation curve showing different species present during aggregation. (B) Sequence of hIAPP and 3A-hIAPP showing the oligomeric β-sheet with the black arrow. Alanine substitutions in 3A-hIAPP sequence are shown in red, marked with black arrow. (C) High-resolution structure of 3A-hIAPP oligomer with position of all three alanine substitutions shown in red. The position of Ser-20 is indicated in black with arrows. (D) Pulse shaping scheme showing pump pulses and the stationary probe pulse. Time delay between pump pulses is denoted as *t*_1_ and between the pump and probe pulse is denoted as *t*_2_.

Human islet amylin polypeptide (hIAPP) is a 37-residue polypeptide (Figure 1B) that forms transient oligomeric intermediates and ultimately aggregates into amyloid plaques implicated in type 2 diabetes. These transient oligomers are cytotoxic and thought to permeabilize the cell membrane, cause inflammation, and interfere with receptor-mediated processes.^1,2,4,15–17^ We recently reported an atomic structural model of oligomers formed by a version of hIAPP that has three alanine substitutions at Leu-12, Phe-15, and Asn-31, that we call 3A-hIAPP.^6^ These substitutions slowed aggregation from hours to days, enabling structural characterization by high-resolution 2*D*/3D NMR spectroscopy (sequence and substitutions are shown in Figure 1). Extensive control experiments were performed to assess the validity of this model. These alanine substitutions did not perturb cytotoxicity, oligomer size distribution as measured by cross-linking, or the morphology according to TEM.^6^ Moreover, a β-sheet previously identified using isotope labeling and two-dimensional infrared (2D IR) spectroscopy located in the famous Phe-23 to Leu-27 region of the sequence, was recapitulated in the alanine-substituted model.^18,19^ The resulting atomic structure of the dimer created from the NMR data is shown in Figure 1C.

The derived structure consists of 4 structural features: a loop constrained by a disulfide bond between Cys-2 and Cys-7, N-terminal α-helices that span from Ala-5 to Val-17 for one strand and extending to Asn-21 in the other (Figure 1C), followed by a kink that ends at Phe-23, and a parallel β-sheet spanning Phe-23 to Ser-28 (“FGAILS” region). This oligomeric model is notable for structural and biophysical reasons. The FGAILS β-sheet correlates with the likelihood of a species to develop amyloid plaques and type 2 diabetes; it is only observed in species that develop type 2 diabetes.^20^ The N-terminal sequence that spans the helices are conserved across species and is likely the recognition sequence of IAPP.^21,22^ The helices are amphipathic, which promotes helical bundles and plays a role in membrane binding and aggregation.^21^ The kink that lies in the middle of the helix and the β-sheet is the site of a serine-to-glycine (at Ser-20) hereditary mutation that is associated with the early onset of type 2 diabetes.^23^ Our experimentally derived structural model also has similarities with earlier computationally derived structures,^24,25^ such as that by Ding and co-workers who also predicted a helix-kink-sheet structure of the oligomers, albeit with a more compact tertiary fold.^25^ Overall, the features of the structural model in Figure 1C are consistent with prior biophysical studies and physiological observations.

The atomic model developed from 3A-hIAPP is physiologically relevant only to the extent that its structure and biochemical properties resemble those of hIAPP. In our original study, we presented spectroscopic evidence that the FGAILS β-sheet in our model has a comparable structure as hIAPP. Here, we test the N-terminal helices and surrounding disulfide-loop and kink regions. The helix is of relevance since the alanine substitutions typically promote helix formation,^26,27^ and so Ala-12 and Ala-15 in 3A-hIAPP could artificially extend the length of the helices. To study these regions, we modified our 2D IR spectrometer so that it could collect waiting-time-dependent 2D IR spectra of hIAPP and 3A-hIAPP during aggregation. The waiting time (defined as *t*_2_) is the second delay in a typical 2D IR pulse sequence (Figure 1D). During this time, spectral features decay because of vibrational relaxation thus, modes with shorter lifetimes gets suppressed at longer *t*_2_, which leads to higher-resolution spectra that can resolve hidden spectral features. Previously, our group and others have found that 2D IR spectra collected at longer *t*_2_ reduces contributions from random coil features in spectra of hIAPP fibrils.^28,29^ At *t*_2_ = 1 ps, spectra were shown to be less congested, which allowed improved resolution of β-sheet features.^29^ These experiments were performed on fully aggregated hIAPP fibrils that were no longer kinetically evolving, so signals could be averaged without time constraints. However, for oligomers, spectra must be collected while aggregation is occurring, making rapid data collection imperative. At concentrations typically used in our 2D IR experiments (0.1–1 mM), monomers assemble to oligomers within minutes, followed by aggregation into amyloid fibrils within hours (Figure 1A).^6,19^ During this process, the relative concentrations of these 3 species (monomer, oligomer, and fibril) evolve continuously, as do the resulting 2D IR spectra as shown in Figure 1A. Thus, the speed at which the spectra can be measured determines the accuracy with which the structural kinetics can be monitored. Previous studies on this subject relied on mechanical stages that increment the waiting-time delay by physically moving two retroreflector mirrors, which is a slow process (typically, tens to hundreds of milliseconds per step).^28,29^ By generating the waiting time electronically using a pulse shaper rather than a mechanical stage, we could update the pulse sequence shot-to-shot, allowing us to interweave 2D IR pulse sequences so that multiple-waiting-time spectra could be continuously measured throughout aggregation. Similar pulse shaping strategies have been previously implemented in other nonlinear spectroscopy.^30,31^ The resulting spectra are much less congested and reveal features created by the N-terminal α-helix and the neighboring loop and kink regions. The spectra are nearly identical between hIAPP and 3A-hIAPP, which strongly supports the relevance of our oligomeric structural model and establishes a rigorous workflow that could be applied to generate and test atomic structural models of oligomers associated with early-onset type 2 diabetes, IAPP oligomers from other species, and oligomers from other amyloid diseases.

## Methods

### Preparation of Protein Sample

hIAPP samples were prepared using FMOC microwave-assisted solid-phase peptide synthesis following previously published procedures,^19^ briefly summarized here. For the synthesis, we use polystyrene resin (PALPEG-PS) and FMOC-protected amino acids. Synthesized polypeptides were cleaved using an acidic cleavage cocktail containing trifluoroacetic acid:thioanisole:ethanediol:anisol in a 90:5:4:1 (v:v:v:v) at 37 °C for 30 min in microwave-assisted cleavage system (CEM). After cleavage, peptide is precipitated and washed in cold ether. To form the Cys-2–Cys-7 disulfide bond, the polypeptide is dissolved in DMSO for 24 h followed by a 50% dilution with 20% acetic acid for 12 h. Separate syntheses were performed for each isotope labeled samples. We labeled different residues in hIAPP with ^13^C^18^O. Labeled amino acids were prepared using ^18^O exchange on ^13^C labeled FMOC-protected amino acids (purchased from Cambridge Isotope Laboratories) using ^18^O water and anhydrous 1, 4 dioxanes containing 4 M HCl. Two separate labeling experiments are discussed. One, with labeled Phe-23 to Ala-25, and other with labeled Val-32, Gly-33. hIAPP is purified using two rounds of reversed-phase HPLC system that uses a C18 column (XSelect CSH, Waters). We use a gradient of two buffers—A: 0.045% (v/v%) HCl in water and B: 80% acetonitrile, 20% water and 0.045% HCl (v/v%). HPLC is run at 1% per minute until 45% Buffer B was reached. After the first round of purification, peptides were dissolved in HFIP and diluted with 4x water and repurified using same gradient. MALDI-TOF (Bruker Ultraflex) was used to characterize and assess the purity of the hIAPP samples. Samples were quantified using Nano Drop (Thermo Fischer) using 280 nm absorbance with an extinction coefficient of 1490 M^–1^ cm^–1^ and FTIR (Nicolet iS10, Thermo Scientific). In addition to unlabeled and labeled hIAPP, we synthesized a version of hIAPP, referred to as 3A-hIAPP, that has alanine substitutions at Leu-12, Phe-15, and Asn-31 (Figure 1B). These three alanine substitutions extend the aggregation time of the hIAPP oligomers while keeping the structure intact, enabling us to obtain a high-resolution structure of 3A-hIAPP using NMR spectroscopy. All samples were prepared by reconstituting the lyophilized peptide to 500 μM in 50 mM tris buffer prepared in D_2_O. To minimize extent of aggregation, we did not perform any stirring. The sample was quickly placed between two CaF_2_ windows using 75 μm spacer such that the dead time between reconstitution and data acquisition remained less than 2 min.

### 2D IR Spectroscopy and Pulse Shaping

2.5 μJ mid-infrared pulses centered at 6200 nm having a pulse width of 75 fs at 10 kHz repetition rate were generated using a commercial Yb:KGW laser system and OPA (Carbide and Orpheus, from Light conversion). A 75/25 ZnSe beam splitter is used to create two mid-IR laser beams, with the higher intensity beam sent through a pulse shaper that is set in a 4f-geometry to generate the pump pulses. An arbitrary waveform generator (AWG, from Signatec) created the optical masks in the pulse shaper and was used to shape the spectrum and phase of the pump pulse to create 2 pulses and scan the coherence time delay (*t*_1_) and waiting-time delay (*t*_2_). Waiting-time delays were created by applying a linear phase to the waveform. The maximum double pulse delay that can be generated at high fidelity with our pulse shaper when configured with 75 g/mm gratings is 5 ps at 5.5 μm. This delay must include both the *t*_1_ and *t*_2_ delays. The maximum coherence delay was set to *t*_1_ = 2.5 ps so that a waiting times up to 2.5 ps could be measured; we report spectra collected at *t*_2_ = 0, 0.6, and 1.2 ps, which is within the relevant *t*_2_ range for proteins.^28,29,32^ All spectra were collected using a four-frame phase cycling scheme, and *t*_1_ was incremented in 40 fs steps, thereby requiring 256 combinations of pulses for each 2D IR spectrum. The pulse sequence for each waiting time was interwoven so that each *t*_1_ delay sequence has its own *t*_2_ delay. We made three such sequence for acquiring three *t*_2_ delays corresponding to 0, 0.6, and 1.2 ps, one after the other before data collection for the next loop starts. Spectra were measured at 3 min interval and representative 2D IR spectra shown were averaged over 9–12 min window. We note that the diffraction efficiency of the pulse sequence depends on the *t*_2_ delay due to the finite resolution of the shaper, which can be corrected (see Figure S1). As noted above, many more than 3 waiting times can be collected as the AWG card holds up to 10× more memory than what we utilized in this study. Pump and probe beams are focused on the sample cell using 90° parabola to create a spot size of ∼60 μm. The probe beam is dispersed onto a 64-channel mercury cadmium telluride (MCT) array detector using 75 g/mm gratings, giving a probe frequency resolution to 4 cm^–1^. Each MCT element is read shot-to-shot (JackHammer, from PhaseTech spectroscopy). 2D IR spectra are generated by a mathematical Fourier transform along *t*_1_ and an optical Fourier transform of the probe pulse to give the frequency axes ω_pump_ and ω_probe_, respectively.

### Modeling of 2D IR Spectra

2D IR simulations of the model oligomeric structure were performed using a MATLAB-based simulation package, Coupled OScillator MOdel Spectrum Simulator (COSMOSS), which is based on vibrational exciton theory.^33^ The local mode frequency was set to 1650 cm^–1^ for the amide I transition, which primarily consists of the carbonyl stretch and some C–N stretch and N–H bend. A combination of the transition dipole coupling (TDC) model and Jansen coupling map was used to calculate coupling constants. TDC was used for long-range interactions, and for estimating short-range coupling, we used the Jansen map.^34^ Diagonal disorders were neglected in presented calculations. In this work, we show the representative 2D IR spectra measured from the most stable conformation obtained from 2*D*/3D NMR and MD simulations.

## Results

### Waiting-Time-Resolved 2D IR Spectra Uncovers the N-Terminus Helices of hIAPP Oligomers and New Cross-Peaks

The aggregation of hIAPP was monitored continuously over 7 h (*T*_agg_). The kinetics of hIAPP aggregation as monitored via the presence of amyloid fibrils using the β-sheet feature (peak I, described below) is shown in Figure 2A. The kinetics are sigmoidal, typical of amyloid nucleation, with a half-rise time of *T*_agg_ = 3.5 h, and lag phase of *T*_agg_ = 2 h. Aggregation is mostly complete by *T*_agg_ = 5 h. During the lag phase, there exists an equilibrium between monomers and oligomers.^6^

**Figure 2:**
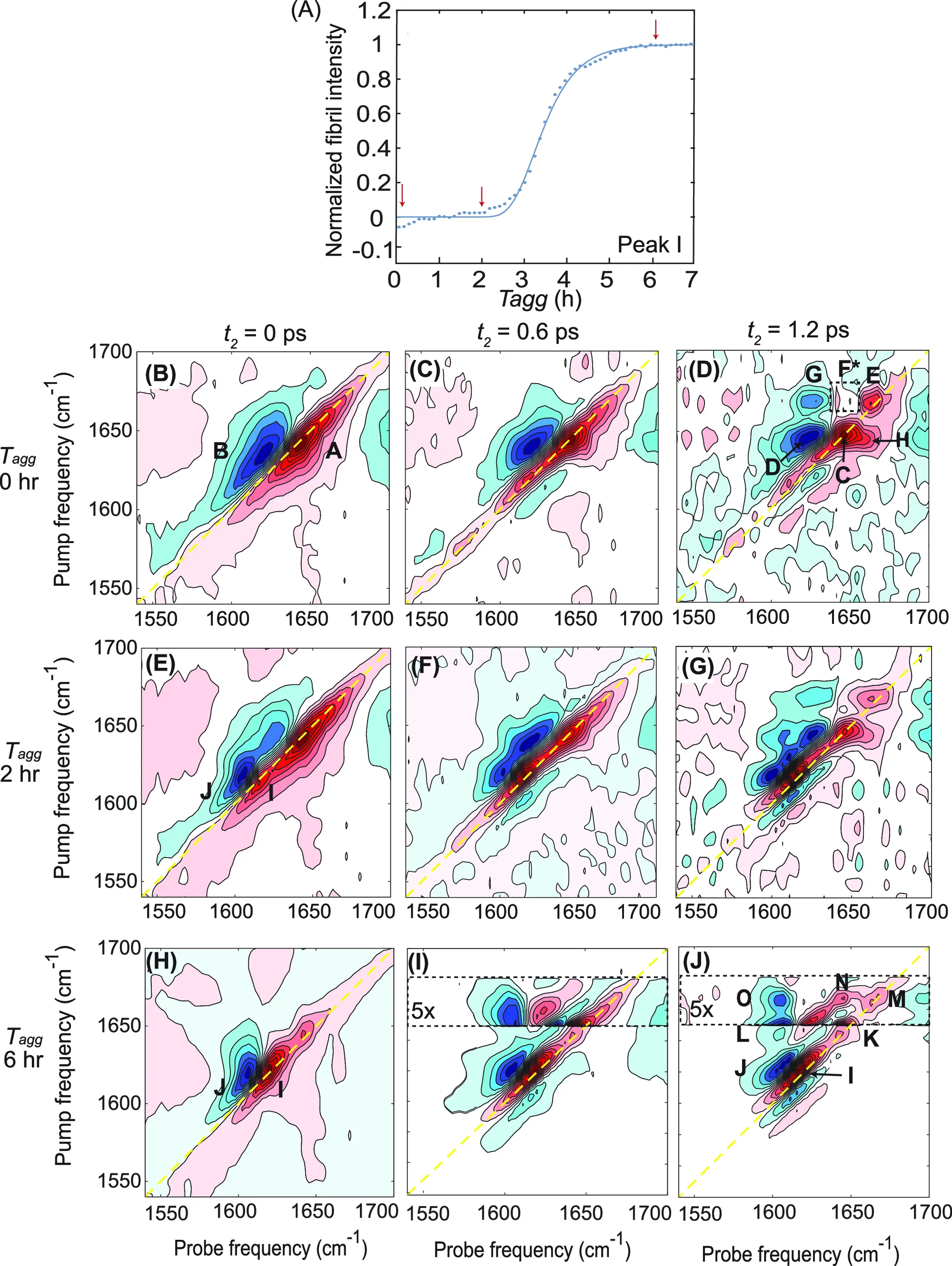
2D IR spectra and aggregation curves of unlabeled hIAPP. The diagonal is shown as the yellow dotted line. (A) Aggregation curve of hIAPP obtained from intensity of fibril peak (peak I). 2D IR spectra of hIAPP at *t*_2_ of 0, 0.6, and 1.2 ps and (B–D) *T*_agg_ of 0 h. (E–G) *T*_agg_ of 2 h. (H–J) At *T*_agg_ of 6 h.

Shown in Figure 2 are representative spectra, at *T*_agg_ = 0, 2, and 6 h, for early lag phase, toward the end of lag phase, and fibril spectra, respectively. Figure 2B–D contains the 3 spectra corresponding to *t*_2_ = 0, 0.6, and 1.2 ps at *T*_agg_ = 0 h. The spectrum at *t*_2_ = 0 ps is broad, centered at 1645 cm^–1^ along the ω_pump_ axis (peaks A and B, in Figure 2B) (Unless noted otherwise, peaks will be referenced by their ω_pump_ frequency and label. Table S1 lists the pump and probe frequencies for each peak) and has no other clearly defined features. This spectrum is typical of a congested spectrum that is dominated by random coil structures. In contrast, the spectrum at *t*_2_ = 1.2 ps, where signal from random coil mostly decayed due to faster vibrational relaxation,^28^ is much better defined (Figure 2d), with at least 4 clearly resolved and sharp features (labeled C, D, E, and G).

Peaks C and D are consistent with the α-helix of the oligomer structure in Figure 1C. We present the following evidence in support of this conclusion: First, peaks C and D have a frequency which is very typical for a helix.^35^ Second, with the increasing *t*_2_, we see a decrease in intensity due to vibrational relaxation, and a frequency shift (overlaid spectra and diagonal slices are provided in Figure S2). The center frequency of peak C is slightly blue-shifted (∼3 cm^–1^) compared to peak A (Figure S2). Similarly, at the end of aggregation, peak K corresponding to the delocalized mode in fibril as observed in previous studies,^29^ is red-shifted by ∼4 cm^–1^ compared to the peak C (see Figure S3). Thus, peak C neither represents random coil nor any fibril mode. Third, the narrow line shape of peak C and D is consistent with an excitonic vibration, i.e., a vibration delocalized over many coupled oscillators.^35,36^ Fourth, the feature arises from oligomeric helices, not monomeric, which we conclude from a comparison to 3A-hIAPP given below. The narrow line shapes suggest a homogeneous oligomer structure, although neither the 2D IR nor the NMR data used to generate the oligomer structure is size-selected.^6^ Additionally, the size distribution does not appear to change during the lag phase, consistent with a stable oligomer structure followed by a nucleating event.^19^ Thus, based on these observations, we conclude that introducing a waiting time of 1.2 ps has decreased the spectral congestion, leaving the spectral signature of a delocalized mode, consistent with the N-terminus α-helices of the oligomer shown in Figure 1C.

The *t*_2_ = 1.2 ps spectra also resolve a second diagonal peak (peak E) and an off-diagonal peak (peak G) both at ω_pump_ = 1667 cm^–1^. Peaks E and G are separated by 36 cm^–1^ along ω_probe_. The off-diagonal peak (peak G) is not an overtone transition, but a cross peak. 2D IR pulse sequences generate pairs of peaks with opposite phases formed by fundamental and overtone transitions. Peak pairs are separated by diagonal anharmonicity that creates an anharmonic shift of about 16 cm^–1^ for amide I vibrations,^32^ which is far too small to explain the 36 cm^–1^ frequency difference between peaks E and G. Instead, we conclude that the peak separation arises from the destructive interference occurring at the position marked F*. The position of F* occurs where a cross peak would be expected between peaks C and E, which we highlight with the dashed box in Figure 2D. Coupling between C and E-mode would produce a positive cross peak that overlaps with the negative overtone of peak E, causing destructive interference. Indeed, simulations using a 2 × 2 coupled matrix shown in Figure S4 illustrate how this type of interference can occur. Additionally, the corresponding cross peak (peak H) below the diagonal, at ω_pump_ = 1647 cm^–1^ shows aggregation kinetics similar to peak E (see Figure S5). Thus, we conclude that the α-helix mode at 1647 cm^–1^ is coupled to the mode at 1667 cm^–1^. In a later section, we use simulations and additional experiments to assign the 1667 cm^–1^ feature to the kink regions of the oligomers with contributions from the disulfide region.

As aggregation proceeds and fibrils form, as shown in Figure 2E–G (*T*_agg_ = 2 h) and Figure 2H–J (*T*_agg_ = 6 h), peaks A and B give way to a peak pair at ω_pump_ = 1620 cm^–1^ (peaks I and J). These peaks are created by the parallel β-sheets of the amyloid fibrils and were used to follow the aggregation kinetics in Figure 2A. By 5 h, aggregation is complete, and all features can be assigned to fibrils. While aggregation proceeds, the helical peaks C and D disappear as the oligomer disappears. Peak E becomes less intense, and its cross peak G to the helix is replaced by the cross peak O with the β-sheet mode. Peak K appears at ω_pump_ = 1643 cm^–1^, which is assigned to a delocalized mode of the fibril β-sheet as previously observed.^29^ The kinetics of peaks N and O are very similar to the fibril kinetics (peak I), confirming that these features arise from the fibril as well (Figure S5).

Thus, introducing a waiting time into the 2D IR pulse sequence decreases spectral congestion, revealing the α-helical mode of the oligomer (peaks C and D) and a high-frequency mode (peak E) that is coupled to the helix (peaks G and H).

### 2D IR Spectra of 3A-hIAPP Are Nearly Identical to hIAPP

Shown in Figure 3 are the results from experiments performed on 3A-hIAPP analogous to those on hIAPP shown in Figure 2. The alanine substitutions placed into hIAPP to create 3A-hIAPP slow fibrillization so that it takes >30 h for 3A-hIAPP to fully aggregate (Figure 3A). Considering the extended aggregation time, we show representative 2D IR spectra at *T*_agg_ values of 0, 18, and 35 h. These points lie at the very beginning, at the end of the lag phase, and after the fibrils have fully formed, respectively, analogous to the time points shown for hIAPP but on a slower time scale.

**Figure 3:**
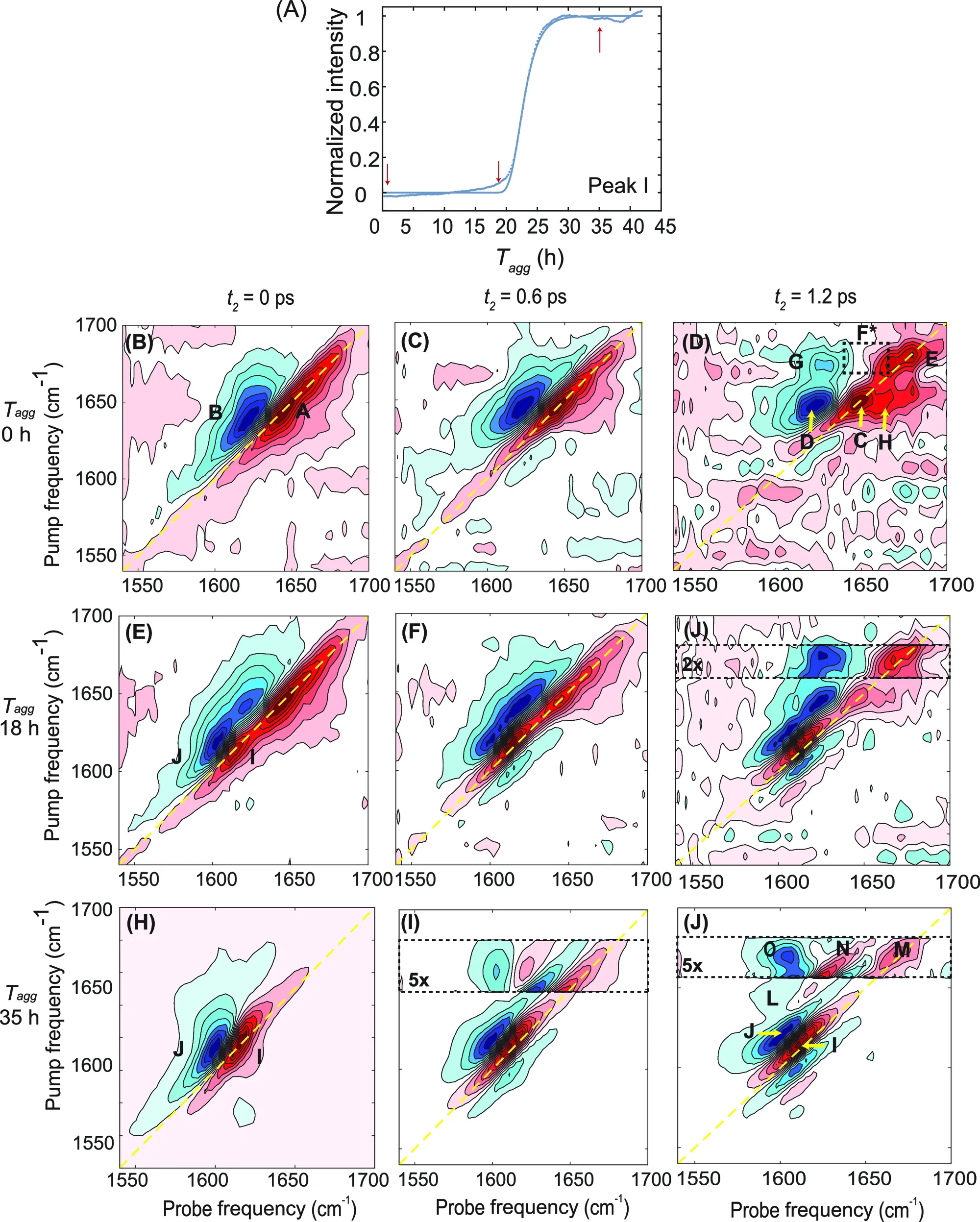
2D IR spectra of 3A-hIAPP and aggregation curve. The 2D IR spectra of 3A-hIAPP looks remarkably similar to hIAPP spectra shown in Figure 2 except with a longer aggregation time. The diagonal is shown as the yellow dotted line. (A) Aggregation curve of 3A-hIAPP obtained from intensity of fibril peak (peak I). 2D IR spectra of 3A-hIAPP at *t*_2_ = 0 ps, 0.6, and 1.2 ps and (B–D) *T*_agg_ of 0 h. (E–G) *T*_agg_ of 18 h. (H–J) *T*_agg_ of 35 h.

The spectra of 3A-hIAPP are remarkably similar to hIAPP. At *T*_agg_ = 0 h and *t*_2_ = 1.2 ps, when 3A-hIAPP is in the oligomeric form, peaks C, D, E, and G are clearly observed. The relative intensities and line widths are also nearly identical (Figure S6). Thus, the 3A-hIAPP oligomer has the same secondary structure as that of hIAPP to within the resolution of our experiments. More details are given below, but that means that the N-terminal α-helix and high-frequency mode at ω_pump_ = 1667 cm^–1^, which we later assign to the kink and loop regions, as given in Figure 1, are structurally correct. Indeed, it was previously established that 3A-hIAPP exists during the lag phase almost entirely in the oligomer structure and so the strong similarity to hIAPP indicates that the *t*_2_ = 1.2 ps waiting-time spectrum eliminates the monomeric features from the spectra, consistent with their largely disordered structure.^6^

We are not particularly interested in the structure of the fibrils because the alanine substitutions were chosen to hinder aggregation, and thus, they are expected to form nonphysiological fibrils. Alanine substitutions replaced bulky side chains to reduce the hydrophobic contacts and alter the steric zippers that are thought to stabilize fibrils.^37^ Even a single-point mutation in hIAPP has been shown to produce alternate fibril polymorphs.^38^ Nonetheless, we include the 2D IR spectra of the fibrils to help highlight the uniqueness of the oligomer features, the effectiveness of sharpening the spectra using waiting-time delays, and how the spectra are utilized to monitor fibrillization.

### Kink Contributes to the High-Frequency Features of the Oligomers

In this section, we systematically investigate and discuss each of these possible assignments for peaks E and G. These two features lie at ω_pump_ = 1667 cm^–1^, which is at the high end of typical values observed for amide I band of proteins. Possible origins for features in this range include (a) side-chain absorbances, (b) the FGAILS β-sheet, (c) the E-mode of the α-helices, and (d) regions of the oligomer with nontypical secondary structures that have large couplings. We note that peak E is strongly coupled to the α-helix when in the oligomeric form, as evidence by the F* region in Figures 2D and 3D, as discussed above. In what follows, we walk through our reasoning supported by experiments and simulations that conclude that peaks E and G are created by nontypical secondary structures in the kink and disulfide-loop regions.

We reached this conclusion from the following arguments:

(a) There are no side-chain absorbances in this high-frequency region in a deuterated solvent. In H_2_O buffer, asparagine, arginine, and glutamine side chains absorb near 1667 cm^–1^, but those side chains absorb at about 1620 cm^–1^ in D_2_O, the solvent used in these experiments.^28,39,40^ Even when these side chains are coupled as occurs, for example, when side-chain “ladders” are formed in amyloid fibrils like in polyQ,^41^ they only reach 1645 cm^–1^. Acidic side chains, aspartic acid and glutamic acid, are not present in hIAPP or 3A-hIAPP (sequence shown in Figure 1A).
(b) Small β-sheets are known to absorb in this frequency range.^42^ Oligomers have a small β-sheet that spans residue FGAILS. Thus, to test if the FGAILS β-sheet is the source of these features, we synthesized hIAPP with ^13^C^18^O labels at residues FGA and performed analogous aggregation experiments. The data are shown in Figure S7. The signal from the labeled FGA residues is shifted to 1570 cm^–1^ (peaks P and Q), but peaks E and G are unchanged and thus not created by the β-sheet. We also note that peaks C and D are also unchanged, consistent with the assignment of these two peaks to the helices.
(c) The E-mode of α-helices are much weaker in intensity and lie higher in frequency compared to the A-mode discussed above.^43,44^ The frequency difference between the two modes can vary anywhere from a few cm^–1^ up to 10 cm^–1^ in ideal helices, oftentimes these two modes are indistinguishable from each other.^45,46^ In any case, observed frequency is 15 cm^–1^ higher than the A-mode with comparable intensity. Thus, we rule out this possible assignment.
(d) That leaves noncanonical secondary structures as the remaining assignment. To that end, we systematically simulated portions of the oligomer structure looking for high-energy features (simulation results from individual segments are shown in Figure S8). We identified three regions that could explain the high frequency. First, across the entire C-terminus, from residues Phe-23 to Tyr-37, the only location that created a higher frequency feature was Val-32 and Gly-33 (Figure S8f). To experimentally test this region, we synthesized and measured ^13^C^18^O-labeled hIAPP at Val-32 and Gly-33, which lie in the middle of the C-terminus which would disrupt the expected nearest neighbor coupling. Those labels did not alter peaks E and G (Figure S9). Moreover, these residues are not coupled to the helices and so do not explain the cross-peaks G and H. Thus, the C-terminal region is not responsible for the 1667 cm^–1^ peaks.

Shown in Figure 4 are the simulations of the two other regions that produce high-frequency features, namely, the kink and disulfide-loop regions. Unlike Val-32 and Gly-33, these spectra exhibit cross-peaks to the α-helix, explained Peak E is created by dihedral angles that produce large positive coupling constants. Indeed, it is well established that kinks and turns have high-frequency modes around 1670 cm^–1^.^47^

**Figure 4:**
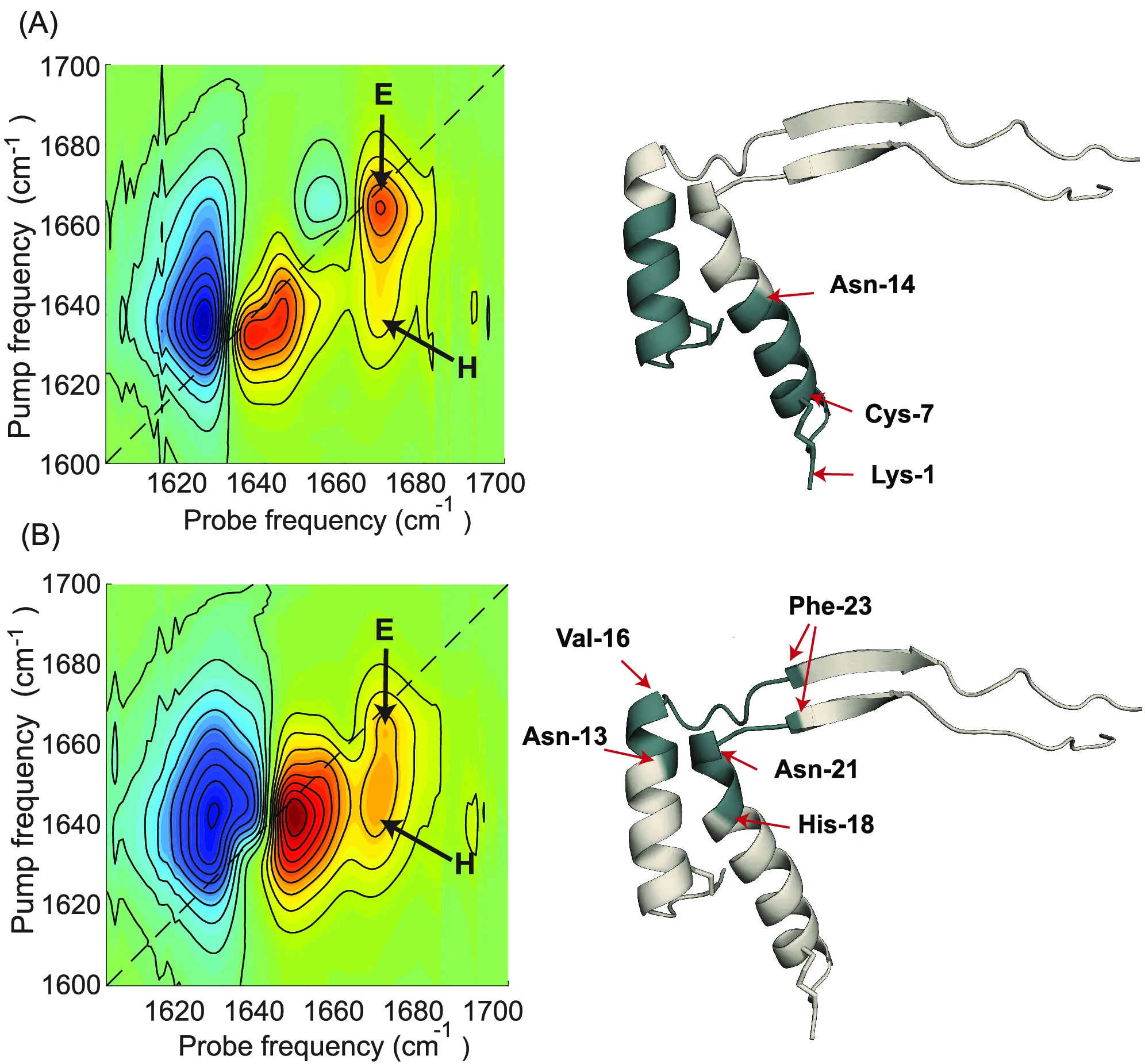
Simulated 2D IR spectra of disulfide and kink region of oligomer. (A) Simulated 2D IR spectra of residues Lys-1 to Cys-7 on the C-terminal constituting the disulfide bond, and helical residues until Asn-14. Simulated segment is shown in green. (B) Simulated 2D IR spectra of kink residues, Val-16 and Asn-21 to Phe-23, and neighboring helix residues. Simulated segment is shown in green.

To experimentally tested the kink region, we measured spectra of S20G hIAPP. Glycine mutations are common in regions that connect secondary structure elements because glycine is more flexible, so it is known to stabilizes kinks or turns.^48^ S20G is a hereditary mutation in hIAPP that is associated with early-onset type 2 diabetes.^23^ The 2D IR spectrum of S20G hIAPP exhibits the same helix peaks C and D and the same high-frequency peaks E and G. However, the intensity of peaks E and G are much larger, eclipsing the α-helix peak (Figure 5B), whereas in hIAPP and 3A-hIAPP, peaks E and G were half as intense as the α-helix peak (Figure S6). Thus, peaks E and G at 1667 cm^–1^ are definitively assigned to the kink region.

**Figure 5:**
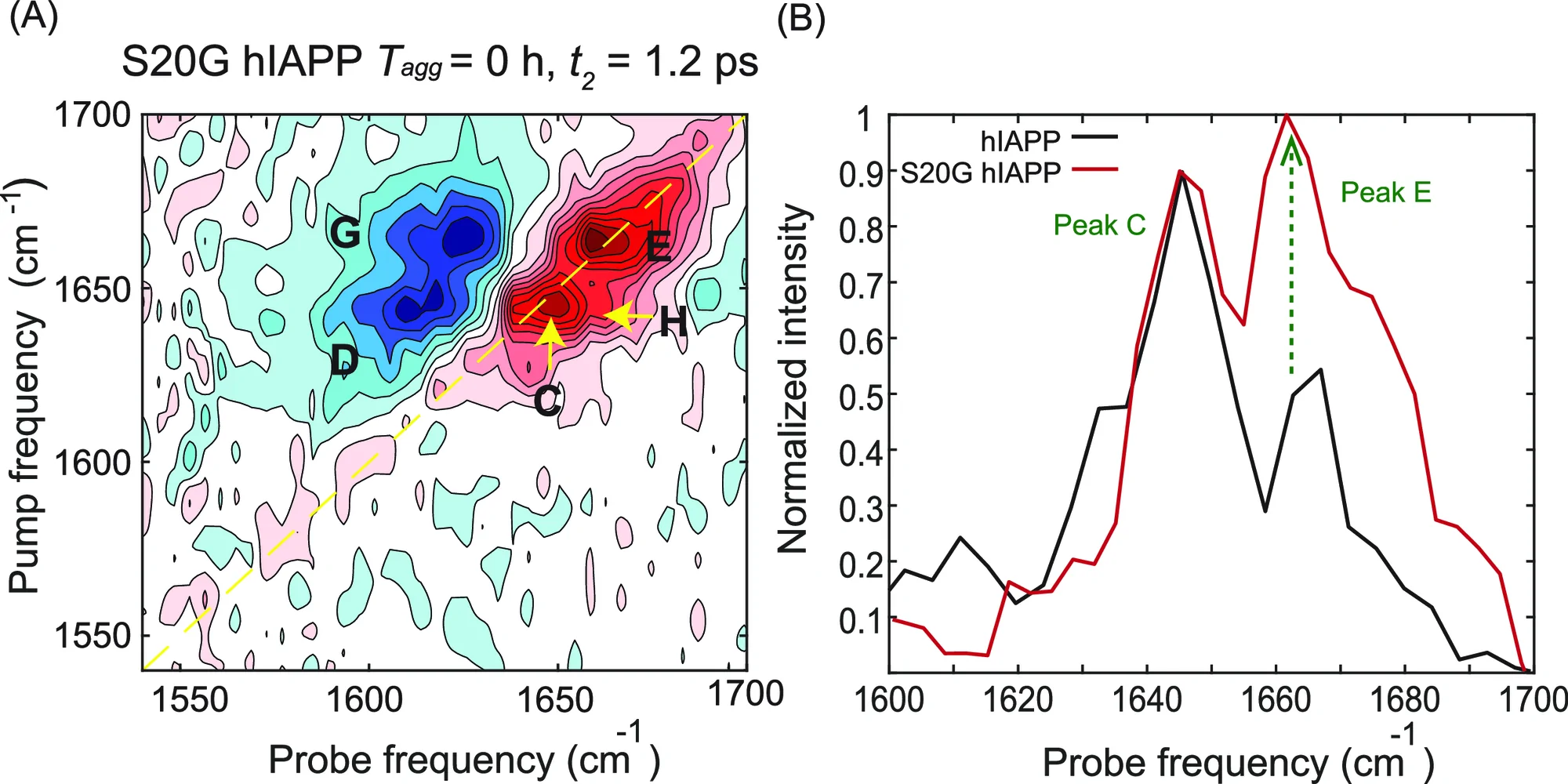
2D IR spectra of S20G hIAPP at the start of aggregation and comparison of diagonal slice with hIAPP. (A) 2D IR spectra of S20G hIAPP at *T*_agg_ of 0 h and *t*_2_ of 1.2 ps. (B) Normalized diagonal slices of hIAPP and S20G hIAPP. To show a better comparison for peak E intensity, the diagonal slice of hIAPP is scaled such that the intensity of the helix feature matches.

The disulfide-loop region could not be directly tested. Ala-5 is the only residue in that region that is straightforward to isotope-label, but labeling a single isotope is usually insufficient to disrupt secondary structure couplings. We also synthesized C2S and C7S hIAPP to eliminate the disulfide bond, but aggregation occurred within a few minutes, similar to other reported studies.^49,50^ Therefore, we could not observe the oligomeric state. Thus, we can definitively assign peaks E and G at 1667 cm^–1^ to the kink region but cannot eliminate the possibility that residues within the disulfide bond also contribute.

## Discussion

This paper reports data collected with a modified 2D IR spectrometer that incremented waiting-time delays optically rather than mechanically. This technical development allowed us to intertwine several pulse sequences so that multiple 2D IR spectra can be collected nearly simultaneously. Rapid data collection made it possible to collect 2D IR spectra with several waiting time while hIAPP aggregates, thereby measuring amyloid oligomers before they fibrillize. Spectra with a waiting time of *t*_2_ = 1.2 ps resolved the α-helix in the oligomers and uncovered a high-frequency feature that we assigned to the kink region connecting the α-helix to the FGAILS β-sheet. The spectra of hIAPP and 3A-hIAPP are nearly identical. These results have important implications for the structural model of hIAPP oligomers and for the physics of waiting-time experiments.

This study strengthens the argument that 3A-hIAPP is a good structural model for the hIAPP oligomers. The 2D IR spectra of 3A-hIAPP and hIAPP during the lag time are nearly identical for all waiting times. The helix frequencies lie within 3 cm^–1^ of each other (see Figure S10 for overlap of hIAPP and 3A-hIAPP 2D IR spectra), indicating that the length of the 3A-helix is very similar or identical to that of hIAPP. Simulations shortening the helix by just 2 residues create a 3 cm^–1^ frequency shift that is outside the experimental difference (Figure S11). That is important information, because alanine mutations are known to stabilize helices and the alanine substitutions at residues Leu-12 and Phe-15 in 3A-hIAPP reside in the helix. Thus, our data are evidence that those two alanine substitutions do not artificially elongate the helix.

The 2D IR spectra of 3A-hIAPP and hIAPP also exhibit the same high-frequency feature at 1667 cm^–1^. We established that the oligomeric kink residues contribute to this feature. The oligomeric kink region is adjacent to the helix and spans part of the critical 20–29 residue, which is a critical determinant of type 2 diabetes susceptibility.^51,52^ A hereditary mutation having Gly-20 in place of Ser-20 shows faster aggregation and higher cytotoxicity.^23^ Thus, knowing the structure of this region is critical. Our data support the structural model of hIAPP generated from 3A that is shown in Figure 1C as being a rigorous construct for the N-terminal helices and adjacent turn and possibly loop regions.

It is also interesting to find that the waiting-time experiments resolve the helical eigenstates of the oligomers. Previously, we reported that amyloid fibrils of hIAPP exhibited sharp β-sheet features at *t*_2_ = 1.0 ps which is reproduced here.^29^ We attributed that observation to longer lifetimes of amyloid β-sheets than disordered protein regions. Now seeing a similar observation but for helices, means that 2D IR spectra with *t*_2_ > 1.2 ps will be a useful tool to study decongesting spectra. There are two factors that can help explain the physics behind longer lifetimes of different hIAPP features. One factor is that the lifetimes of random coil regions of proteins are shorter than helices and β-sheets because they are more strongly impacted by solvation dynamics.^29^ Usually, disordered regions of proteins have more solvent exposure, which changes the vibrational dynamics as observed in experiments and simulations.^53,54^ Lifetimes are more difficult to calculate than line shapes, but it would reason that frequency fluctuations would also enable faster intra- and intermolecular vibrational relaxation. That being said, the oligomer is small enough that all residues will be solvent exposed to some degree, which may be why the FGAILS β-sheet, which is small, is not enhanced in the waiting-time experiments here. The other factor is that excitonically delocalized vibrations might have intrinsically longer lifetimes than localized modes.^28^ Delocalization over many eigenstates might decrease overlap with any single state and cause the lifetimes to be longer. Hence, a fast lifetime of any single local mode would collapse the delocalized wave function. We do not believe that there is a compilation of experimental data in the literature that is sufficient to address these questions for proteins nor do we believe that there is a theoretical study that addresses this question directly. It would be useful to have a systematic understanding of the origins. Nonetheless, it is clear from our results that waiting time delays is a very useful tool for decreasing congestion and studying hidden structural features during hIAPP kinetics.

## Conclusions

The decongested 2D IR spectra of the hIAPP reported here are identical to an alanine-substituted version called 3A-hIAPP. Two of the alanine substitutions were made at the N-terminus, so as not to perturb potential helical structure, but it was unknown whether the alanine substitutions altered the helicity of the N-terminus. By generating the waiting time in our 2D IR pulse sequences with a pulse shaper, we were able to measure back-to-back waiting-time 2D IR spectra during aggregation. At waiting-time delays of >1.2 ps, the spectra become much better resolved because the random coil features that caused spectral overlap and congestion in the 2D IR spectra have decayed, leaving the excitonic features created by well-defined secondary and tertiary structures. The spectra resolve the helix and a high-frequency feature created by the disulfide-loop and the kink residues. These features are identical in hIAPP and 3A-hIAPP, supporting the physiological relevance of the atomic structure generated from 3A-hIAPP. With these experiments, we now know that both the C- and N-terminal regions are well represented by 3A-hIAPP. That is important because 3A-hIAPP is based on a structural basis for understanding the long-known correlation between the sequence and disease.

Besides validating our current structural model of hIAPP, the experiments presented here complete a procedure to generate new structural models of hIAPP. The workflow is as follows. A promising variant of hIAPP is identified from ThT or other kinetics measurements that has a lag time of at least 15 h at hundreds of micromolar concentrations, necessary for NMR spectroscopy and represent the physiological concentrations of hIAPP when it is secreted from the pancreatic islets.^55,56^ 2D IR spectroscopy is performed to test the similarity of the variant oligomer to the wild-type oligomer in as many locations as practically possible. For example, we previously tested for the critical FGAILS β-sheet^6^ and here, we tested for the N-terminal helices, and the kink and possibly the disulfide-loop residues. Biochemical assays should also be performed, including cytotoxicity. If all of those pass the test, then 2*D*/3D NMR experiments along with constrained molecular dynamics can be used to produce the atomic structure. With this procedure now in place, it can be applied to obtain models for other amyloid oligomers. Similarly, IAPP oligomers in other diabetic mammals, or of the hereditary mutant S20G hIAPP in humans. Solving a series of IAPP structures could enable new insights into the atomic features that ultimately impact β-cell loss and insulin loss in type 2 diabetes.

## Supplementary Material



## Notes

Additional figures show delay accuracy, overlap of 2D IR spectra and diagonal slices of hIAPP and 3A-hIAPP spectra, simulation results, aggregation curves showing intensity changes of peaks, 2D IR spectra of ^13^C^18^O labeled VG and FGA residues, and a table describing peak positions and assignments. (PDF)
