# Supplementary material for: Decongested 2D IR Spectra Reveal N‑Terminal Helices in Amyloid Oligomers Associated with Type 2 Diabetes
Source: J Am Chem Soc. 2026 Sep 21;148(39):42772–81. doi: 10.1021/jacs.6c16754 (PMC13651530; doi:10.1021/jacs.6c16754)
Supplement: Supplementary file 1 [file ja6c16754_si_001.pdf]

Decongested 2D IR spectra reveal N-terminal helices in amyloid oligomers associated with type 2 diabetes

Shivani T. Shivani[1], Josée Maurais[1], Brynn E. LeMasters[1], Harrison J. Esterly[1], Martin T. Zanni[1*]

[1]Department of Chemistry, University of Wisconsin-Madison, Madison, WI, United States

SI materials

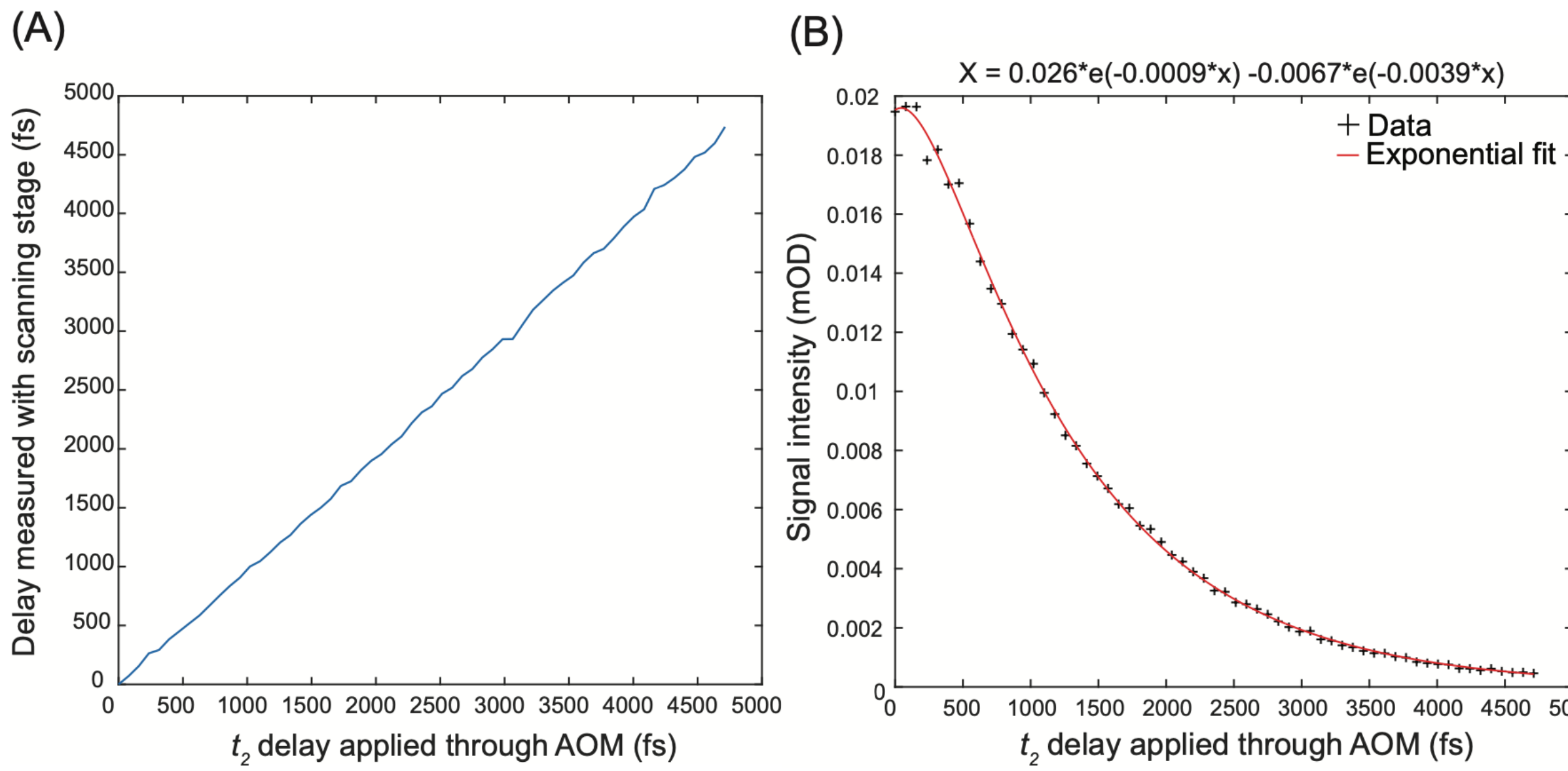

Figure S1: Accuracy of delays generated with AOM using transient absorption (TA) spectra (a) Showing AOM generated delays have a high accuracy. Delays were applied with AOM, and maximum intensity of TA was recovered by scanning stage that indicate t_2 of 0. The resulting plot is linear (b) Signal intensity of the t_2 of 0 ps *i.e.*, TA maxima show an exponential decay in intensity with applied higher t_2 delays. This fit can be used to scale the intensity for measuring spectral diffusion.

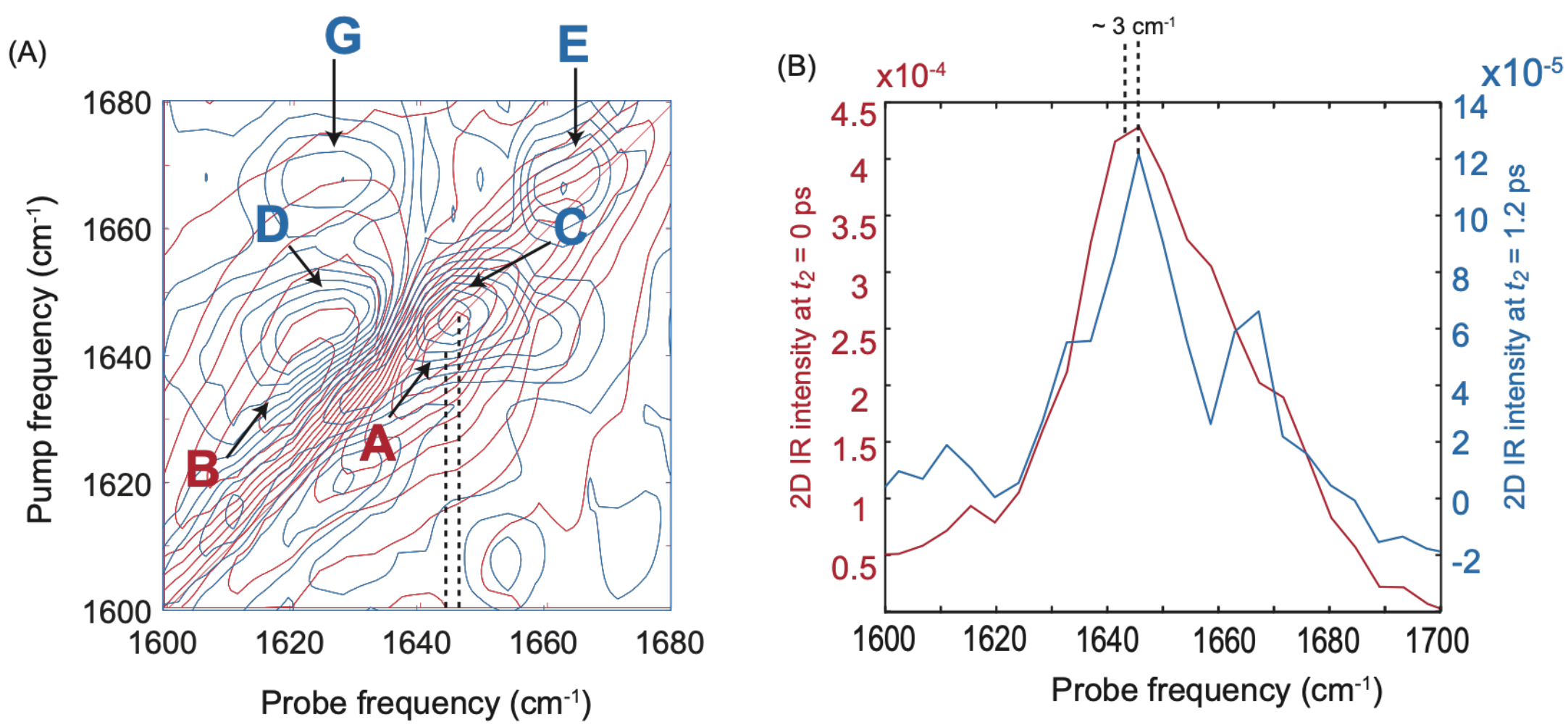

Figure S2: Overlayed 2D IR and diagonal slices of congested and decongested spectra. (a) Overlayed 2D IR spectra of hIAPP at T_{agg} = 0 hr and t_2 = 0 and 1.2 ps showing changes in the peak maxima position. (b) Diagonal slices of the and t_2 = 0 and 1.2 ps are shown in red and blue respectively. 2D IR spectra are taken from Figure 2 (b and d).

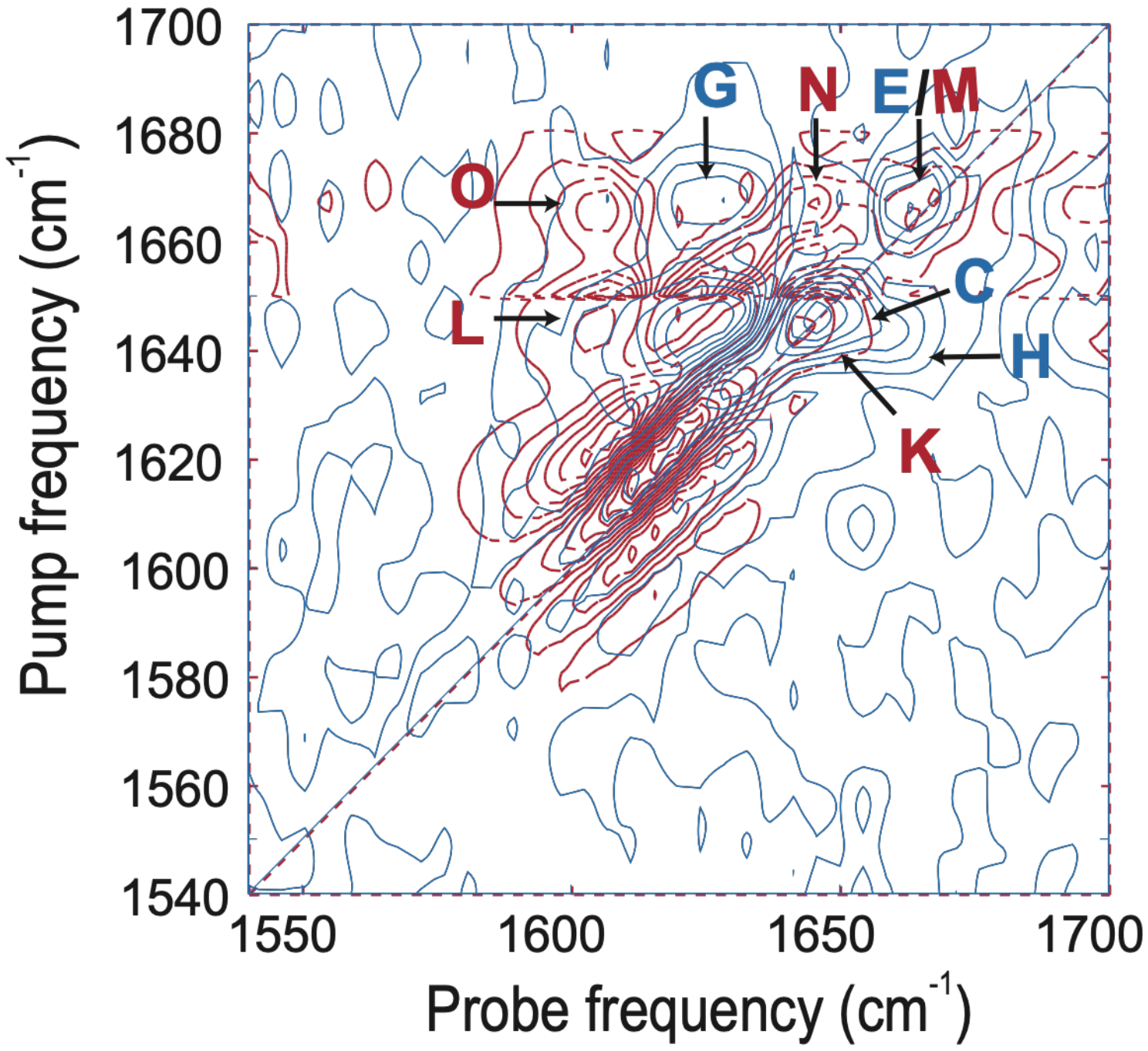

Figure S3: Overlayed 2D IR spectra of unlabeled hIAPP in the lag phase (T_{agg} = 0 h) shown in blue and fully aggregated spectra in red at t_2 = 1.2 ps. The labels for each oligomeric and fibril peak are also shown in respective colors.

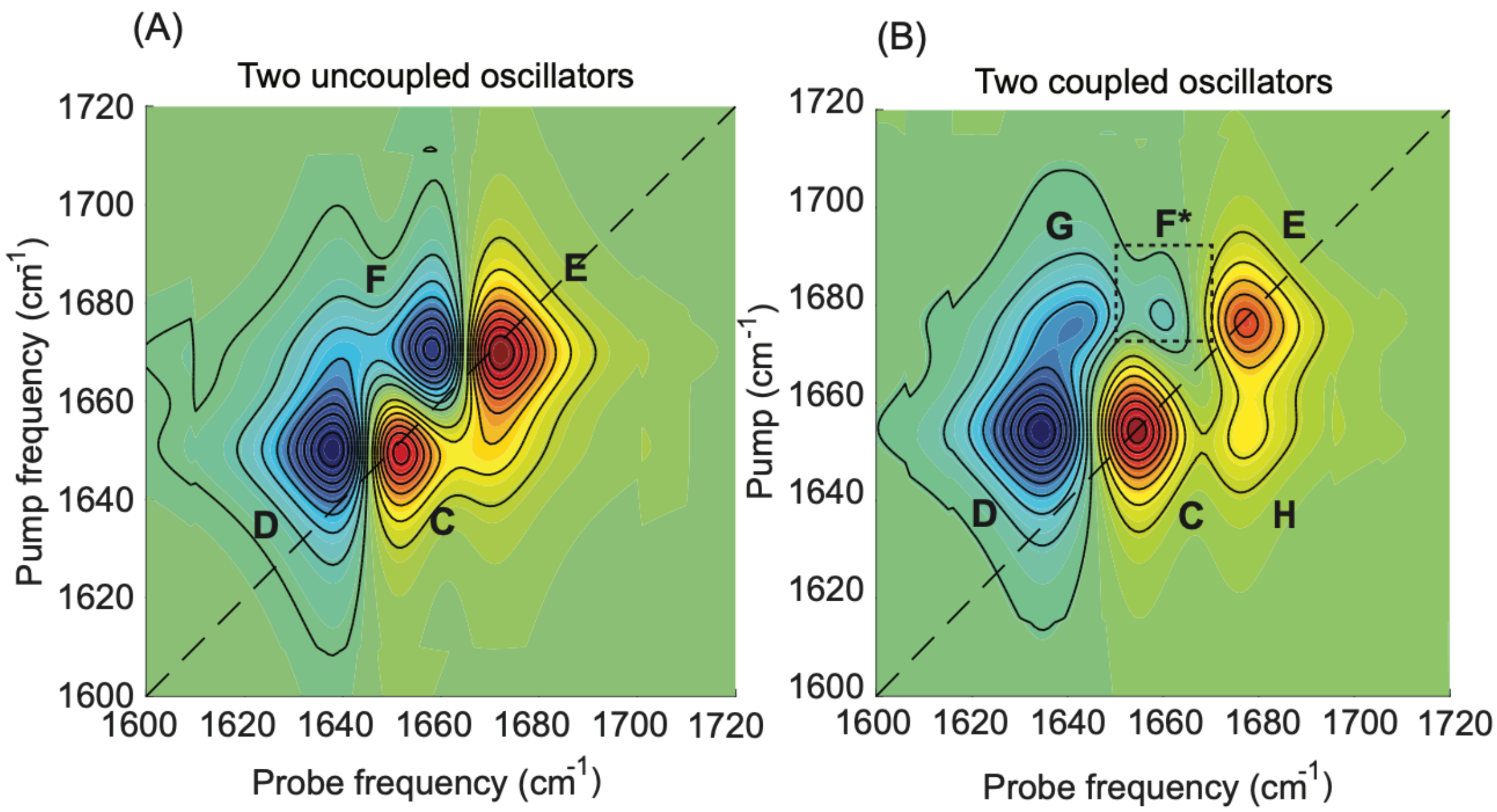

Figure S4: Simulated 2D IR spectra of two carbonyl oscillator having frequency of 1650 and 1670 cm^{-1} separated by 3.5 Å and having angles of ϕ, Ψ, θ of 0°, 0°, 55° and 0°, 0°, -55° respectively (a) with a coupling of 0 cm^{-1} (b) with a coupling of 5 cm^{-1} using Nearest Neighbor and TDC models.

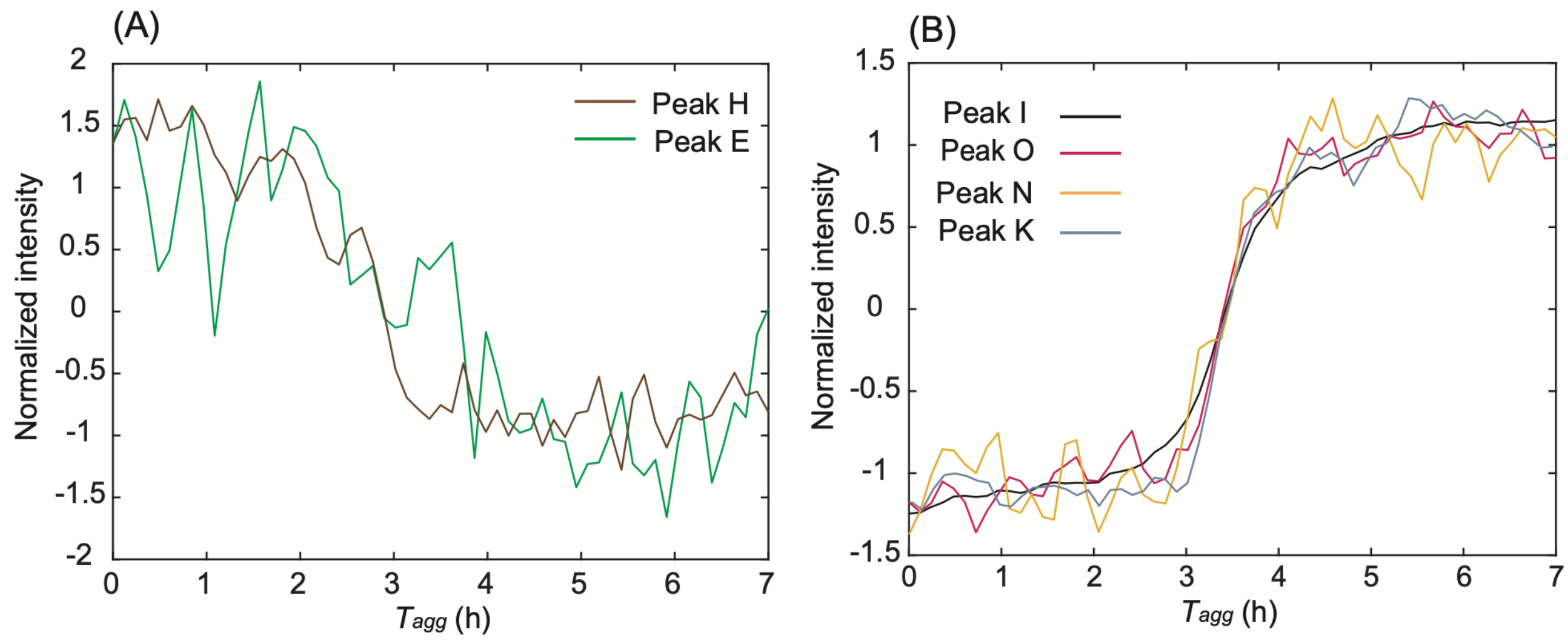

Figure S5: Aggregation kinetics of different peaks at t_2 = 1.2 ps. (a) Peaks H and E correspond to oligomers as decrease with fibril formation. (b) Peaks I, O, N, K increases in intensity as fibril formation starts. The absolute intensity of peak O was plotted since it appears as a negative cross-peak feature.

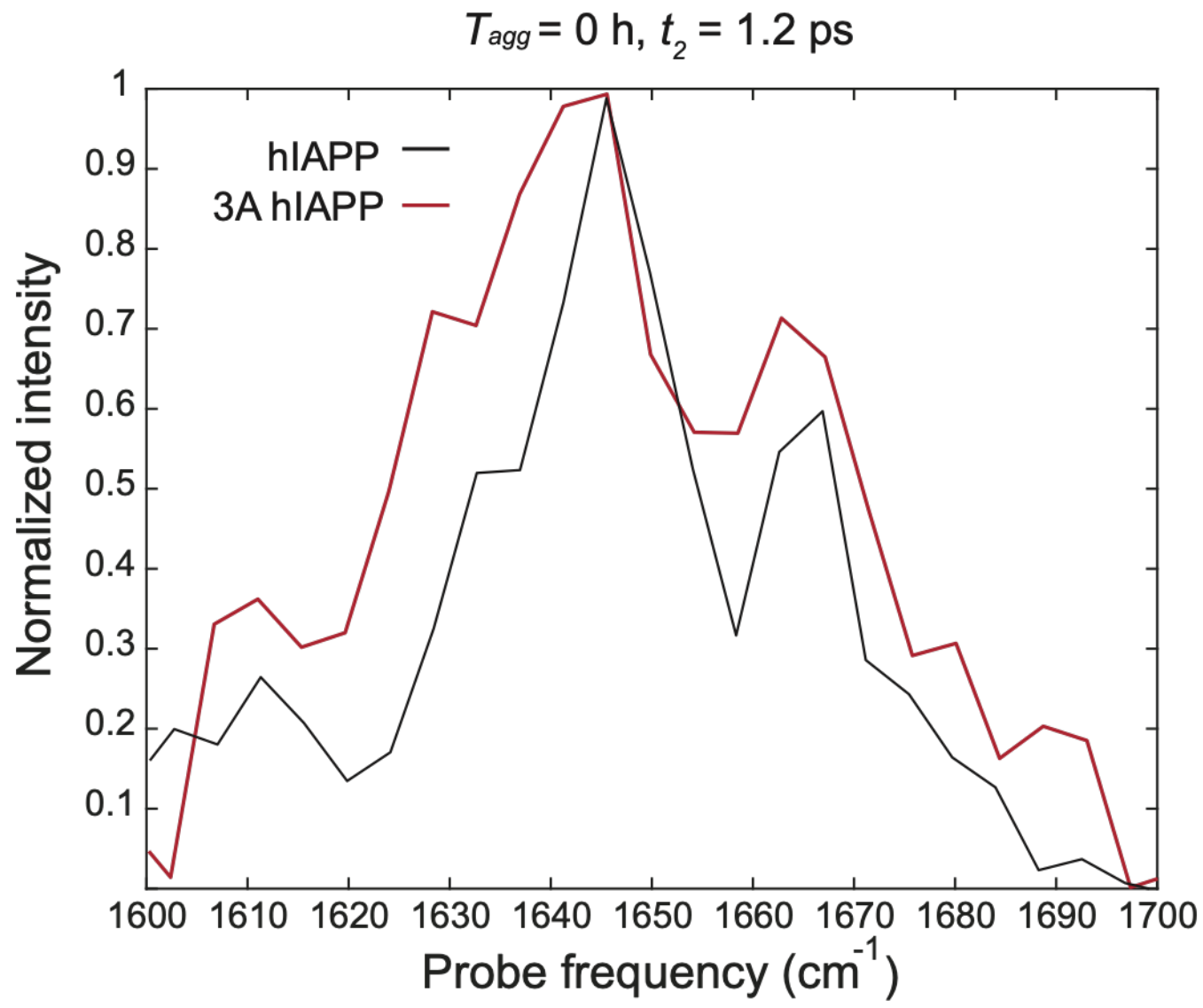

Figure S6: Diagonal slice comparison of hIAPP shown in black and 3A-hIAPP showing in red at T_{agg} = 0 h, t_2 = 1.2 ps. The intensity of the helix region is higher than the high frequency feature around 1667 cm^{-1}.

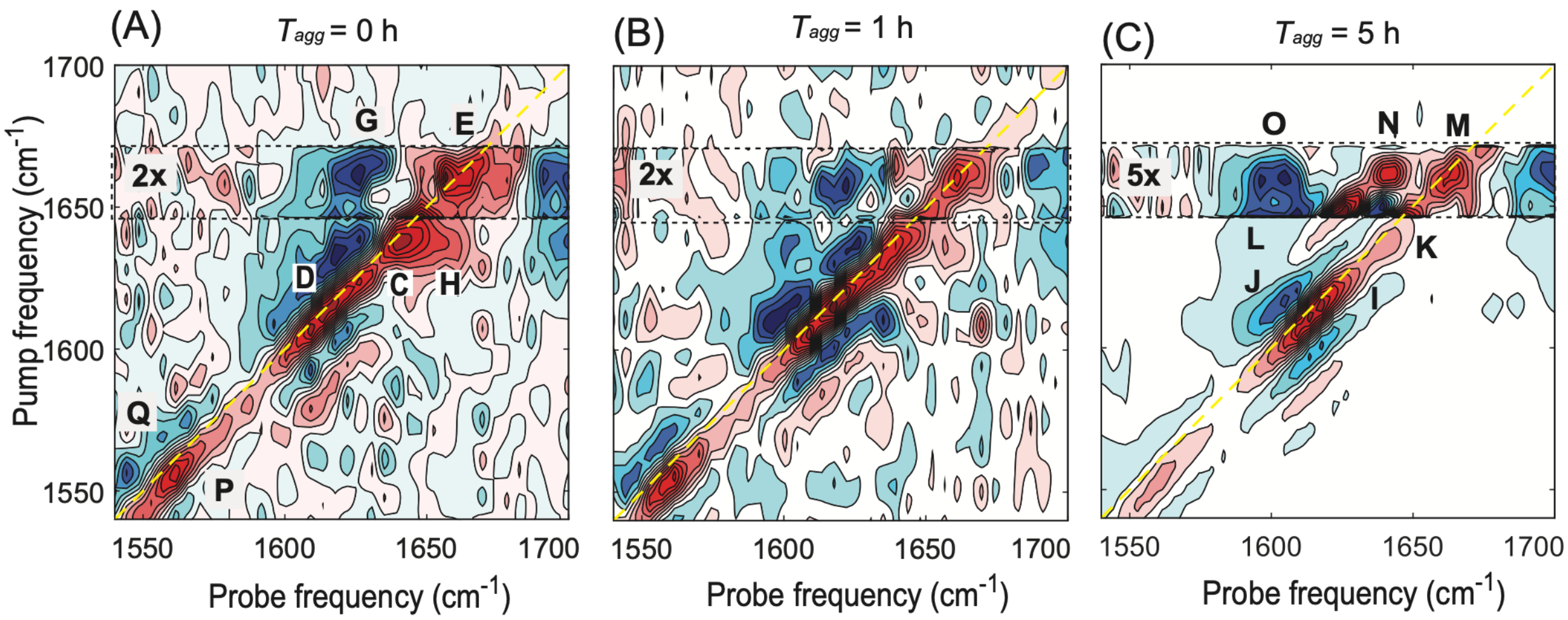

Figure S7: FGA labeled hIAPP 2D IR spectra. (a) In the lag phase, at T_{agg} = 0 hr (b) At the end of the lag phase, T_{agg} = 1.5 hr (c) After aggregation is fully completed, T_{agg} = 5 hr.

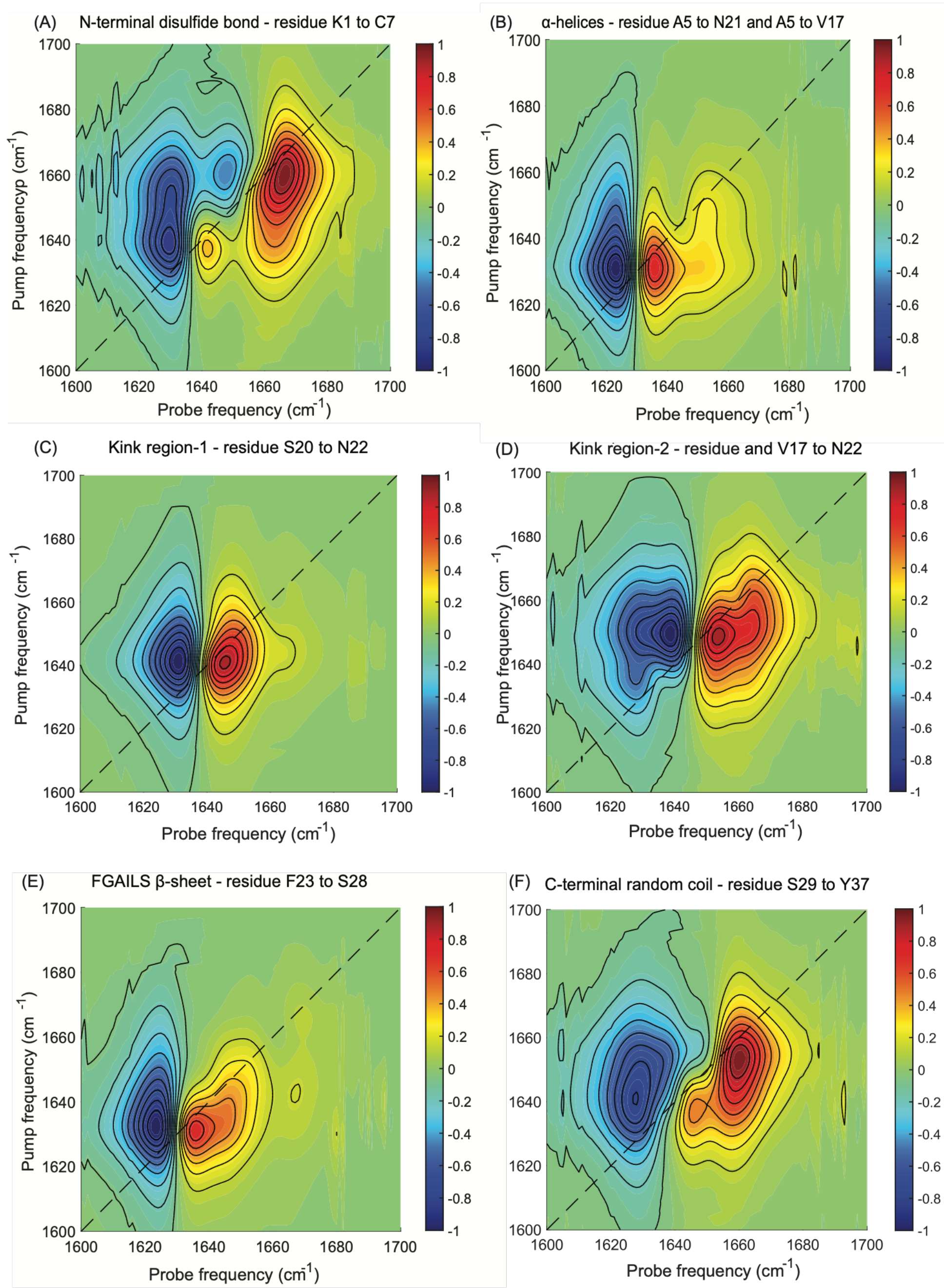

Figure S8: Simulated 2D IR spectra of different secondary structures in 3A-hIAPP oligomeric model (a) N-terminal disulfide bond region comprising of residues Lys-1 to Cys-7 – part of these residues lie in a helix so positive coupling to those helical residues is clearly seen. (b) α-helix region having residues Ala-5 to Asn-21 and Ala-5 to Val-17 – this region only shows a negative coupling. (c) Kink region-1 having residues Ser-20 to Asn-22 (d) Kink region-2 having Val-17 to Asn-22. (e) FGAILS β-sheet having residues Phe-23 to Ser-28 showing negative coupling. (f) C-terminal random coil having residues Ser-29 to Tyr-37.

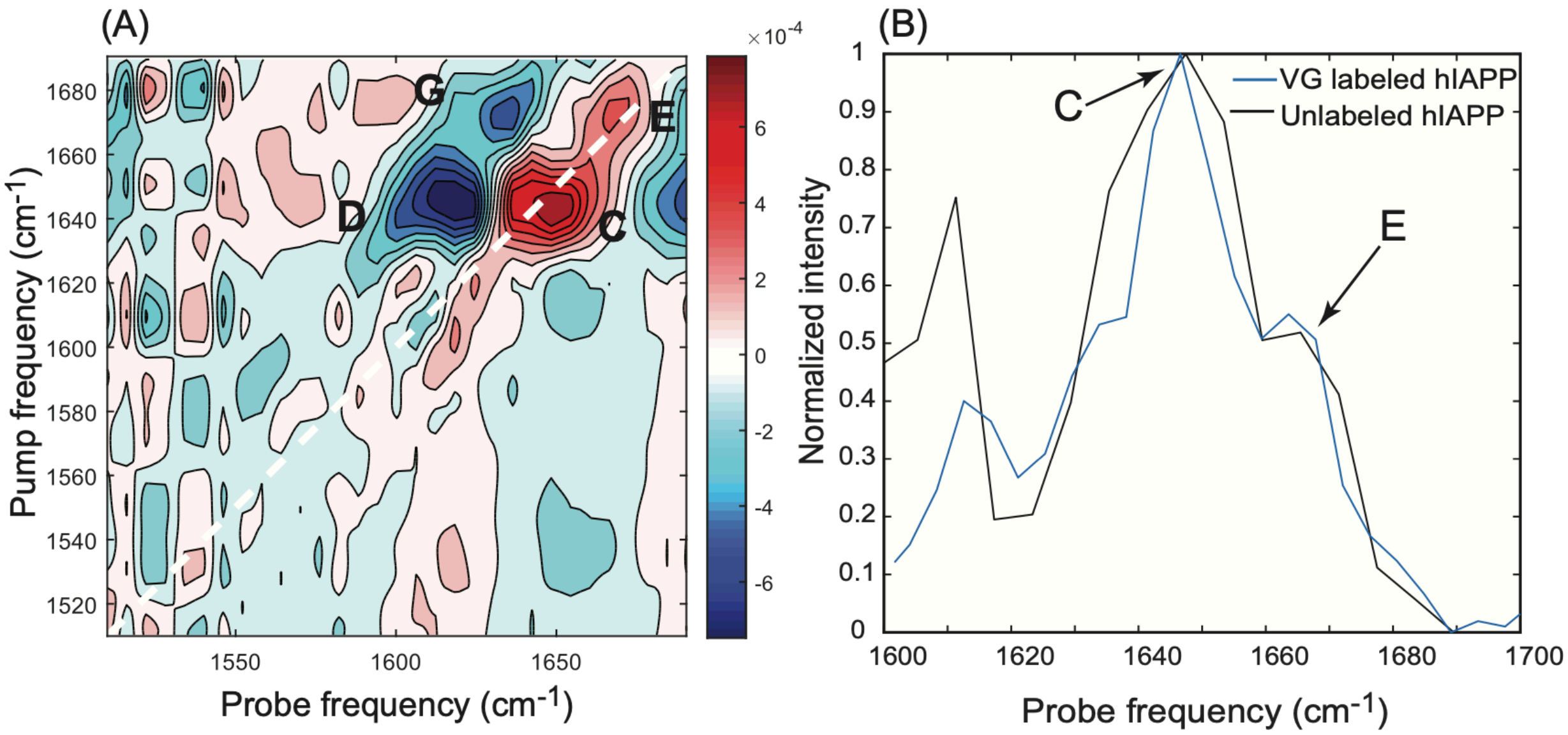

Figure S9: 2D IR spectra of $^{13}C^{18}O$ labeled V32G33 hIAPP showing the α-helix and high frequency feature in the lag time and comparison of unlabeled hIAPP and VG labeled hIAPP

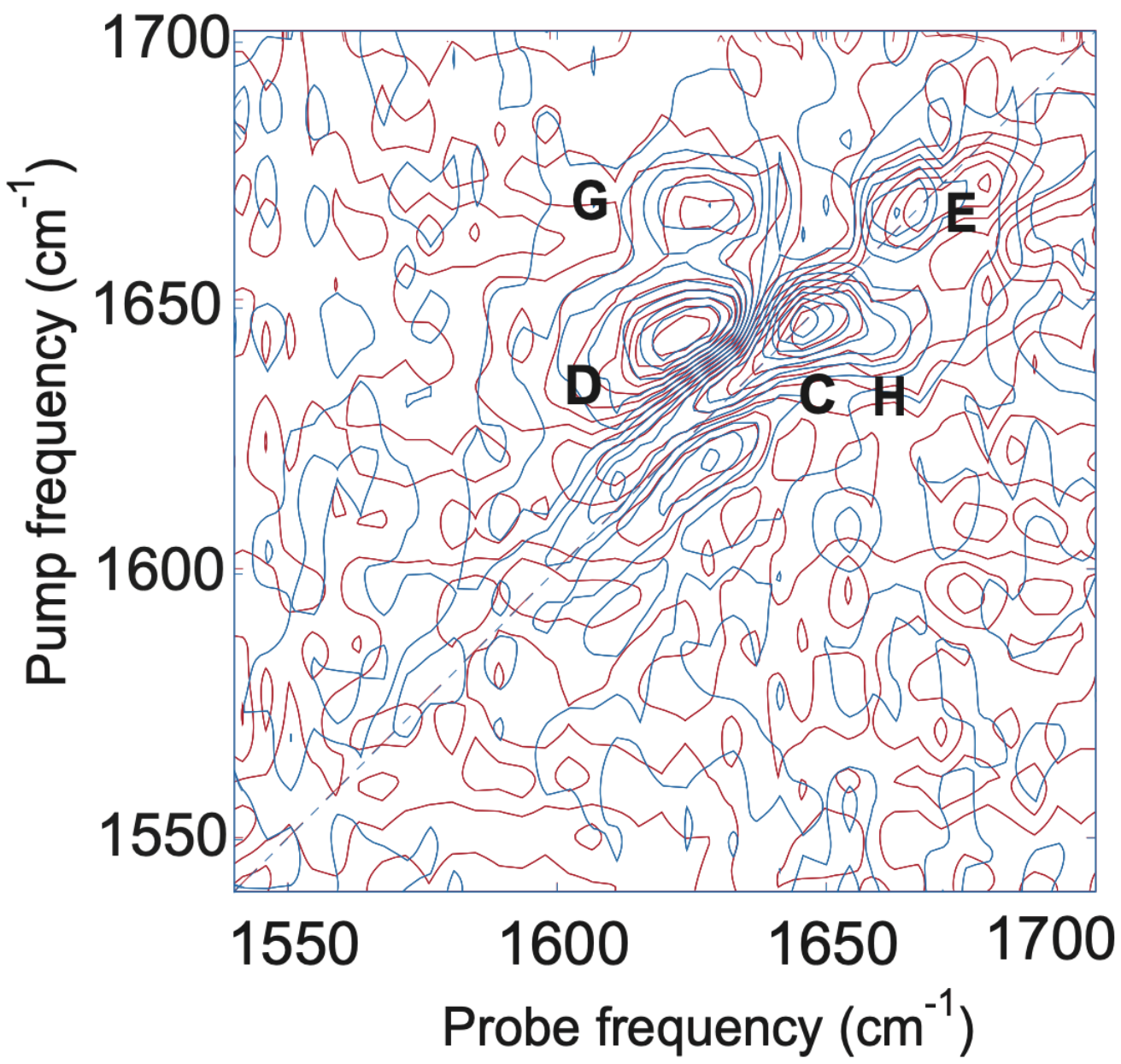

Figure S10: Overlayed 2D IR spectra of unlabeled hIAPP in the lag phase (T_{agg} = 0 h, t_2 = 1.2 ps) shown in blue and unlabeled 3A-hIAPP spectra in the lag phase (T_{agg} = 0 h, t_2 = 1.2 ps) shown in red. Spectra were taken from figure 2d and 3d.

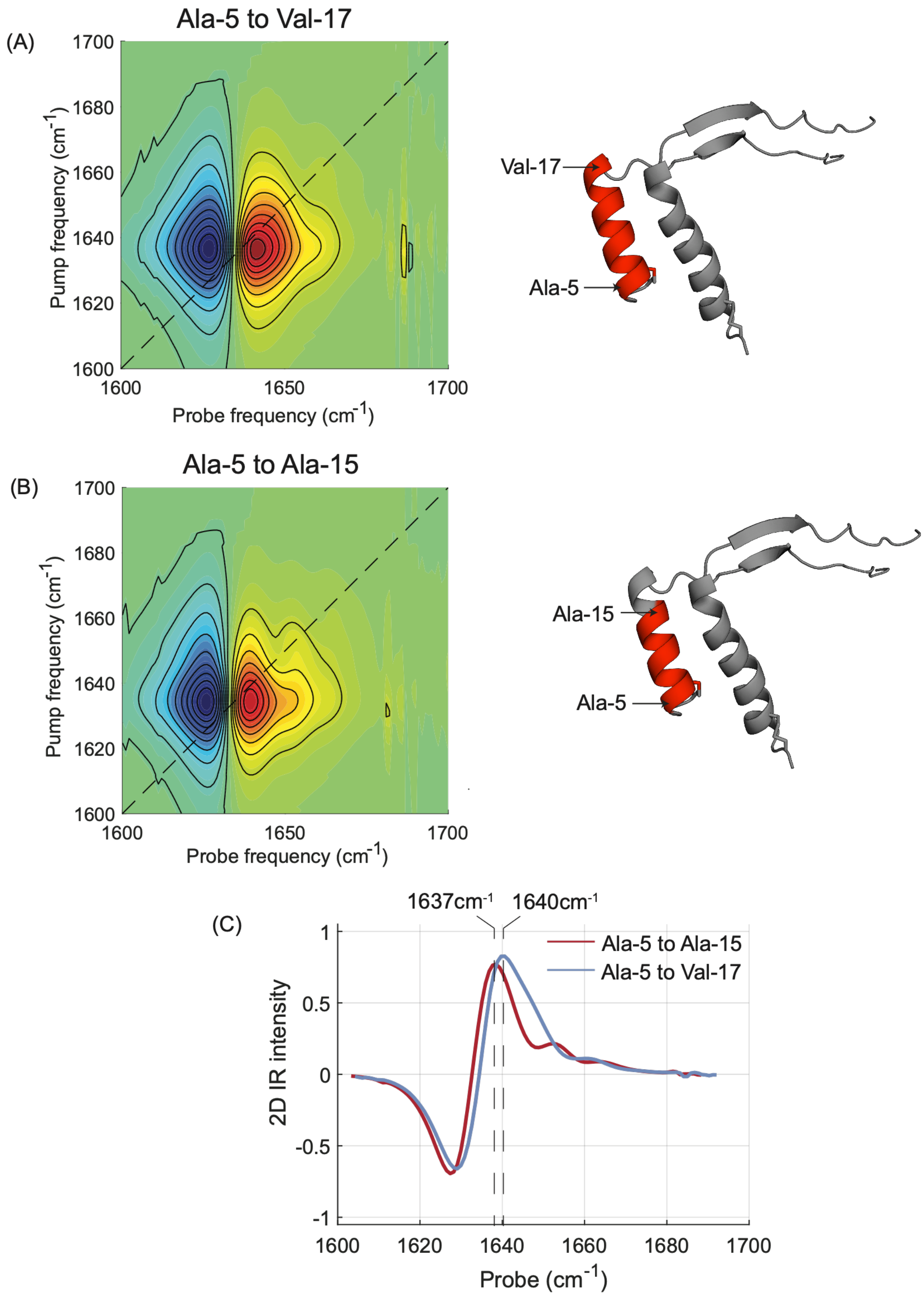

Figure S11: Simulation results from full length helix and helix shortened by 2 residues. (a) Simulation results from full length helix spanning from Ala-5 to Val-17 (b) Simulation results from a shortened helix spanning from Ala-5 to Ala-15. (c) Diagonal slice comparison of (a) and (b) showing a 3 cm^{-1} difference.

**Secondary structure**	**Probe frequency (*ω_3, in cm^{-1}*)**	**Pump frequency (*ω_1, in cm^{-1}*)**	**Peak assignment**
Random coil	1647	1647	A (and B)
Oligomeric alpha helix	1647	1650	C (and D)
Oligomeric kink/turn	1662	1667	E (In fibril, turn into M) (and F*)
Fibril β-sheet	1620	1620	I (and J)
FGA-labeled oligomeric β-sheet	1570	1565	P (and Q)
Fibril random coil	1641	1643	K
Cross peak between random coil and oligomeric kink/turn	1628<br>1662	1667<br>1644	G<br>H
Cross peak between fibril β-sheet and peak M	1606	1667	O
Cross peak between fibril random coil and fibril β-sheet	1606	1643	L
Cross peak between fibril random coil and peak M	1645	1667	N

Table S1: 2D IR Peak (having a frequency resolution of ± 4 cm^{-1}) and frequency assignments of the secondary structures in hIAPP. In case of peak pairs, the diagonal feature (fundamental transition) frequency is discussed, and overtone peak is mentioned in parenthesis as they represent the same secondary structure.
